# Supplementary material for: Hybrid Models and Biological Model Reduction with PyDSTool
Source: PLoS Comput Biol. 2012 Aug 9;8(8):e1002628. doi: 10.1371/journal.pcbi.1002628 (PMC3415397; doi:10.1371/journal.pcbi.1002628)
Supplement: Text S4 — Complete source code for the PyDSTool package (version 0.88.120504). Includes API documentation and help files linking to web pages. This file is identical to the current public release on Sourceforge.net. (ZIP) [file pcbi.1002628.s004.zip › PyDSTool/html/PyDSTool.Toolbox.optimizers.step.quasi_newton_step.DFPNewtonStep-class.html]

xml version="1.0" encoding="ascii"?


PyDSTool.Toolbox.optimizers.step.quasi\_newton\_step.DFPNewtonStep


| Home | Trees | Indices | Help | PyDSTool |
| --- | --- | --- | --- | --- |

Package PyDSTool ::
Package Toolbox ::
Package optimizers ::
Package step ::
Module quasi\_newton\_step ::
Class DFPNewtonStep

[hide private]

[frames] | no frames]

# Class DFPNewtonStep

source code

```
object --+
         |
        DFPNewtonStep
```

---

The Davidson-Fletcher-Powell Quasi-Newton step


|  |  |
| --- | --- |
| Instance Methods | [hide private] |

|  |  |
| --- | --- |
| \_\_init\_\_(self, hessian\_approx)  Construct a DFP module | source code |

|  |  |
| --- | --- |
| \_\_call\_\_(self, function, point, state)  Computes a gradient step based on a function and a point | source code |

**Inherited from `object`**:
`__delattr__`,
`__getattribute__`,
`__hash__`,
`__new__`,
`__reduce__`,
`__reduce_ex__`,
`__repr__`,
`__setattr__`,
`__str__`


|  |  |
| --- | --- |
| Properties | [hide private] |

**Inherited from `object`**:
`__class__`


|  |  |
| --- | --- |
| Method Details | [hide private] |

|  |  |
| --- | --- |
| \_\_init\_\_(self, hessian\_approx)  *(Constructor)* | source code |

Construct a DFP module

- hessian\_approx is an approximation of the hessian around the starting
  point

Overrides: object.\_\_init\_\_

  


| Home | Trees | Indices | Help | PyDSTool |
| --- | --- | --- | --- | --- |

|  |  |
| --- | --- |
| Generated by Epydoc 3.0.1 on Fri May 4 15:24:09 2012 | http://epydoc.sourceforge.net |
